# Genetic Variations and Antibiotic-Related Adverse Events

**DOI:** 10.3390/ph17030331

**Published:** 2024-03-02

**Authors:** Nicola Principi, Kyriakoula Petropulacos, Susanna Esposito

**Affiliations:** 1 Università degli Studi di Milano, 20122 Milano, Italy; nicola.principi@unimi.it; 2 Amici del Bambino Malato Onlus, 41121 Modena, Italy; ptrkrk63m68f257v@gmail.com; 3 Pediatric Clinic, Department of Medicine and Surgery, University Hospital of Parma, 43126 Parma, Italy

**Keywords:** antibiotics, antibiotic prescription, antibiotic-related adverse events, genetic variants, pharmacogenomics, pharmacokinetics

## Abstract

Antibiotic-related adverse events are common in both adults and children, and knowledge of the factors that favor the development of antibiotic-related adverse events is essential to limit their occurrence and severity. Genetics can condition the development of antibiotic-related adverse events, and the screening of patients with supposed or demonstrated specific genetic mutations may reduce drug-related adverse events. This narrative review discusses which genetic variations may influence the risk of antibiotic-related adverse events and which conclusions can be applied to clinical practice. An analysis of the literature showed that defined associations between genetic variations and specific adverse events are very few and that, at the moment, none of them have led to the implementation of a systematic screening process for patients that must be treated with a given antibiotic in order to select those at risk of specific adverse events. On the other hand, in most of the cases, more than one variation is implicated in the determination of adverse events, and this can be a limitation in planning a systematic screening. Moreover, presently, the methods used to establish whether a patient carries a “dangerous” genetic mutation require too much time and waiting for the result of the test can be deleterious for those patients urgently requiring therapy. Further studies are needed to definitively confirm which genetic variations are responsible for an increased risk of a well-defined adverse event.

## 1. Introduction

Antibiotics have been incredibly effective in enhancing health outcomes in humans. With the introduction of these drugs in clinical practice, many once deadly bacterial infections have been effectively treated in the last 70 years, with a significant reduction in morbidity and mortality [1,2]. However, to maximize and maintain these benefits, the prescription of antibiotics must be carefully considered. The use of these drugs is associated with the development of a number of relevant microbiological and clinical problems that can minimize or cancel the antimicrobial efficacy of the prescribed drugs. The abuse and misuse of antibiotics is the most significant cause of the emergence of microbial resistance to commonly prescribed antibiotics, with a progressive reduction in their clinical efficacy, the reemergence of problems regarding the treatment of some bacterial diseases and the need for the development of new antibacterial agents [3]. Moreover, the administration of antibiotics can be associated with the development of short- and long-term adverse events that may be clinically relevant and lead to the need for medical intervention, hospitalization, admission to the intensive care unit and, although rarely, patient death [4,5].

Antibiotic-related adverse events are common in both adults and children, although they are more common among pediatric patients, probably due to the larger use of these drugs in the first years of life. A study carried out in the USA involving mainly adults showed that, in 2013–2014, about 16% of the Emergency Department (ED) visits for adverse drug events were associated with a previous antibiotic prescription [6]. Of these, about 45%, mainly old people, required hospitalization. In children, studies have found that antibiotic-related adverse events cause approximately half of all medical visits for drug-related medical problems and that about 40% of these involve children aged ≤2 years [7,8].

Knowledge of the factors that favor the development of antibiotic-related adverse events is essential to limit their occurrence and severity. The abuse and misuse of antibiotics [9], together with the prescription of unlicensed and off-label drugs [10,11], are among the most important causes of adverse event development, especially in children. In this case, the implementation of carefully planned stewardship programs can be effective in reducing the incidence of adverse events [9,12]. Antibiotic dosages are calculated on the basis of the pharmacokinetic and pharmacodynamic characteristics of each drug. Any modification of the absorption, distribution, metabolism and excretion of a drug due to disease, aging or organ immaturity may lead to a significant increase in the risk of antibiotic-related adverse events [13]. Antibiotic dosages that are well-tolerated and safe in healthy adult patients can be dangerous in sick subjects and in younger infants, as the amount of free drug that is able to exert an antimicrobial effect can significantly increase and reach toxic levels. The personalization of drug dosages according to the characteristics of the patient’s disease and their age and maturity can reduce the risk of adverse event development [13,14]. In order to achieve this goal, particularly for drugs that pose the highest risk, specific dosing tables are prepared to help determine the safe and effective dose for each condition and age.

In some subjects, such as children, antibiotics can interfere with tissue development and cause significant adverse events. The damage caused to both the cartilage in weight-bearing joints and the epiphyseal cartilage following the administration of fluoroquinolones [15], as well as the discoloration of permanent teeth following old tetracycline administration [16], are due to the increased sensitivity of the developing tissues to the antibiotic stimuli. These adverse events should be known, and the use of these drugs in at-risk subjects should be avoided whenever possible.

Finally, genetics can condition the development of antibiotic-related adverse events, and the screening of patients with supposed or demonstrated specific genetic mutations may reduce the incidence of drug-related adverse events. Recent studies have shown that the mutation of genes encoding drug-metabolizing enzymes and transporters, genetic variants of some components of the immune system or mutations of mitochondrial genes are potentially associated with significant modifications of drug disposition [17,18]. This can lead to variations in drug clearance, with reduced drug efficacy or accumulation and an increase in the risk of adverse events. Moreover, in some cases, toxic metabolites are formed. Finally, some genetic mutations are associated with an abnormal immune response, leading to specific tissue damage. For several drugs, the association between well-defined genetic mutations and an increase in the risk of adverse events has been definitively ascertained. This has led several institutions, including the Clinical Pharmacogenetics Implementation Consortium (CPIC), to publish genotype-based drug guidelines to help clinicians understand how available genetic test results could be used to optimize drug therapy in individual patients, according to the characteristics and frequency of genetic polymorphisms in the treated populations [19,20,21,22]. Moreover, health authorities have decided that the risk of genetically determined adverse events should be systematically included in the package leaflet of all the drugs for which this information is known. Regarding antibiotics, however, definitive conclusions have been drawn for very few molecules. For many antimicrobial drugs, the risk of genetically related adverse events has not been sufficiently demonstrated. In other cases, the relationship between the development of adverse events and specific mutations is well defined, but the risk is too low and the genetic screening process capable of identifying at-risk subjects is too complex to justify its introduction in clinical practice. Only for aminoglycosides are there sufficient data to suggest that pretherapy screening analysis should be performed; however, this is very difficult to implement.

This narrative review will discuss which genetic variations may influence the risk of antibiotic-related adverse events and which conclusions can be applied in clinical practice. The MEDLINE/PubMed database was searched from 2000 to 30 November 2023 to collect the literature. The search included randomized placebo-controlled trials, controlled clinical trials, double-blind, randomized controlled studies and systematic reviews and meta-analyses. Abstracts were excluded. The following combinations of keywords were used: “genetics” OR “genetic variations” OR “genetic mutations” AND “antibiotics”” OR “antibiotic-related adverse events” OR “penicillins” OR “beta-lactams” OR “macrolides” OR “aminoglycosides” OR “sulfonamides” OR “antitiberculous” OR “linezolid” OR “quinolones”.

## 2. Antibiotics for Which the Role of Genetics in Conditioning Development of Adverse Events Is Definitively Demonstrated

### 2.1. Aminoglycosides

**Figure 1:**
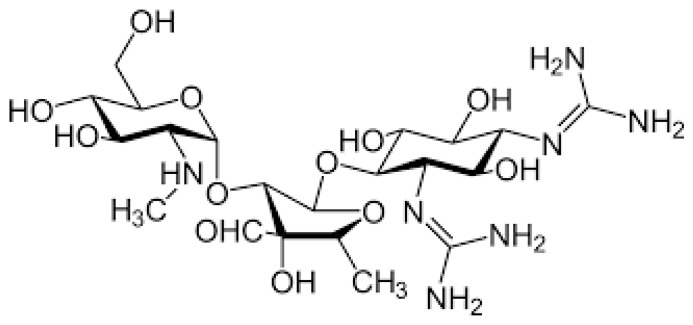
Chemical structure of aminoglycosides.

Aminoglycosides (AGs) are an old class of antibiotics that include neomycin, streptomycin, gentamycin, netilmicin, tobramycin and amikacin among those most frequently prescribed. Figure 1 shows the chemical structure of AGs.

AGs exert concentration-dependent bactericidal activity against several Gram-negative aerobic pathogens and act synergistically with several other antibiotics against some clinically important Gram-positive bacteria, including *Staphylococcus* spp. [23]. The inhibition of bacterial protein synthesis is the main mechanism of action in AGs. They bind to the aminoacyl site of 16S ribosomal RNA within the 30S ribosomal subunit, promote the misreading of the bacterial genetic code and inhibit translocation. This results in error-prone protein synthesis that damages the membrane of the bacterial cell and leads to the death of the infectious agent [24]. Due to their large spectrum of activity, low cost and well-demonstrated persistent clinical efficacy, AGs continue to be frequently prescribed, alone or in combination with other antimicrobials, for the treatment of several suspected or documented life-threatening diseases in both immunocompetent and immunocompromised hosts of any age, including neonates [25]. Unfortunately, the use of AGs is not risk free, as it is frequently accompanied by the development of severe adverse events, among which nephrotoxicity and ototoxicity are the most important. Fortunately, renal damage is reversible; although it can lead to a transient increase in the serum concentration of AGs that favors the development of ototoxicity, it does not lead to permanent alterations in renal function, which returns to normal as soon as AG therapy is suspended. On the contrary, ototoxicity is associated with the development of permanent ear damage that involves the cochlea, the vestibule or both. Cochleotoxicity results in tinnitus and/or sensorineural hearing loss with permanent deafness. Vestibulotoxicity manifests as vertigo, nausea, nystagmus and ataxia. Streptomycin and gentamicin are mainly vestibulotoxic, while amikacin, neomycin and kanamycin are preferentially cochleotoxic. Tobramicin is equally vestibulotoxic and cochleotoxic [26,27].

To exert their cytotoxic effect, AGs must enter the tissue of the inner ear. When systematically administered, these drugs enter the endolymph from the bloodstream and are taken up by inner ear cells via mechanoelectrical transduction channels and apical endocytosis. In the ear cells, AGs bind to the 12S ribosomal RNA subunit of the mitochondrial ribosome and, due to the similarities between mammalian and bacterial ribosomes, interfere with human mitochondrial ribosomes in a similar manner to that in bacteria. When cell respiration is perturbed, the overproduction of superoxide occurs, together with cell apoptosis and the development of ear damage. Moreover, some AGs act on the composition of the otolithic membrane, thus changing its characteristics and causing vestibular damage [27]. Though the development of deafness represents a significant limitation regardless of the patient’s age, this clinical problem is considered a tragic event when it occurs in younger children as it can have a negative impact on language development, literacy, self-esteem and social skills [28]. A recent review of 29 studies carried out from 1975 to 2021, including seven randomized controlled trials, showed that up to 57% of children treated with AGs are at risk of the development of inner ear problems [29].

In most cases, ototoxicity is strictly dependent on the use of higher-than-recommended drug dosages that lead to antibiotic serum concentrations high enough to allow for the penetration of the inner ear. To avoid this risk, in clinical practice, the potential toxicity of AGs is usually handled with therapeutic drug monitoring (TDM). This allows the blood concentrations of the prescribed AG to be maintained in a range that assures maximal clinical efficacy with the lowest risk of developing adverse events [30]. In the case of gentamycin and amikacin, the goal is to maintain drug trough concentrations <2 mg/L in order to avoid potential toxicity [30]. However, ototoxicity can develop even in subjects receiving the recommended AG doses, such as those with severe systemic bacterial infections and ear infections. Animal studies have shown that the activation of toll-like receptor 4 (TLR4) by bacterial lipopolysaccharides potentiates the activity of TRPV1, an important membrane channel that regulates the uptake of AGs into hair cells and thus favors the development of ototoxicity [31]. Moreover, animal studies have shown that ear inflammation increases the uptake of fluorescently tagged gentamicin into hair cells [32]. However, the most important risk factor associated with the development of AG-related deafness in patients receiving recommended AG doses is genetic predisposition. Mutations in the mitochondrial gene *RNR1* (*MT-RNR1*) play a major role in this regard (Table 1).

*MT-RNR1* encodes the 12S ribosomal RNA subunit of the mitochondrial ribosome. It has been supposed that these mutations enhance the similarity of this subunit to the mammalian 16S subunit, thus favoring the attachment of the antibiotic to the ear cells and the development of ear damage [33]. Among the mutations in the *MT-RNR1* gene associated with the development of deafness, the most common is an m1555A>G transition. This is carried by 0.19% (95% confidence intervals [CI], 0.10–0.28) of healthy European children [34] and 0.21% of adults of European descent [35]; it is associated with an almost 100% risk of AG-related hearing loss [36] and has been detected in 5% to 33% of patients with AG toxicity [37]. The *MT-RNR1* gene mutations 1095 T>C and 1494 C>T also play a role in conditioning the development of AG-associated hearing loss, even if the frequency of these genetic variants in the general population and in patients with AG-related deafness is lower than that found for the 1555 A>G variation [37]. The association of many additional *MT-RNR1* variants with AG ototoxicity have been proposed. However, for most of them, there is insufficient evidence to support their association with the risk of AG-associated hearing loss [38]. The clinical relevance of *MT-RNR1* mutations can significantly vary. A wide range of severity, age-at-onset and penetrance of hearing loss has been observed within and among families carrying the *MT-RNR1* gene mutations, suggesting that the phenotypic manifestations of 12S rRNA T mutations can depend on several external factors, such as mitochondrial haplotypes and the type of aminoglycoside [33].

Based on the available data, several institutions and scientific groups have evaluated the need for genetic testing in patients receiving AG therapy. The National Medicines Regulatory Authority in the UK [39] and an international Specialists Pharmacogenomics Advisory Group [37] concluded that genetic testing should not delay urgently needed AG treatment but should be considered before the prescription of AGs in patients with a maternal history of deafness and in those at an increased risk of AG-related adverse events, such as those requiring recurrent or long-term treatment with these drugs; this is considering that the mitochondrial mutations conditioning AG-related hearing loss are relatively rare and that the penetrance of the observed increased ototoxic effect is unknown. Moreover, in patients already diagnosed as carriers of *MT-RNR1* variants, it is recommended that they avoid AGs unless the increased risk of permanent hearing loss is outweighed by the risk of infection without safe or effective alternative therapies. Moreover, in all patients receiving AGs, the continuous monitoring of renal and auditory function, as well as hepatic and laboratory parameters, is recommended [40]. Unfortunately, systematic testing for the most important predisposing mutations remains difficult in clinical practice. Pharmacogenomic-guided antibiotic therapy is limited by the extensive time usually required to obtain genotyping. To overcome this problem, a rapid point-of-care test (POCT) for the m.1555A>G variant has been developed and tested in a group of neonates [41]. A total of 751 subjects with a median age of 2.5 days were recruited. The m.1555A>G variant was genotyped in 26 minutes with 100% sensitivity (95% CI, 93.9–100.0) and specificity (95% CI, 98.5–100.0), without the disruption of routine practice. Three participants with the m.1555A>G variant were identified, all of whom avoided the use of AG antibiotics. Tests like these can facilitate the introduction of pharmacogenomics findings into routine practice and lead to more effective and safe antibiotic therapy in individual patients [41]. 

### 2.2. Beta-Lactams

#### 2.2.1. Amoxicillin–Clavulanic Acid

Amoxicillin–clavulanic acid (AC) is a combination of an antibiotic (amoxicillin) and a suicide inhibitor of bacterial beta-lactamases (clavulanic acid). The inhibition of these bacterial enzymes significantly extends the antibacterial activity of amoxicillin, making the combination effective against a large number of Gram-positive and Gram-negative infections. AC is indicated for the treatment of respiratory tract infections, urinary tract infections and skin and soft tissue infections. Moreover, the unapproved use of this combination in the treatment of several other supposed or documented bacterial infections is common worldwide [42]. In some countries, such as Italy, AC is the most common antibiotic regimen prescribed, especially in children [43]. AC is generally safe and well tolerated, with mild to moderate transient adverse events that are mainly associated with its effect on the gut microbiota. The only severe adverse event that has been reported is idiosyncratic drug-induced liver injury (DILI), which can develop 2 to 45 days after the initiation of therapy [44]. Studies have shown that DILI occurs in about 19.1 per 100,000 persons every year and that AC is the leading cause of this adverse event, with an incidence of 1.7 cases per every 10,000 prescriptions [45]. This frequency may be lower among the pediatric population; however, the data collected in children are very few and do not allow firm conclusions to be drawn in this regard [46]. The clavulanic acid component is considered the true cause of DILI, as the incidence of liver damage in patients receiving amoxicillin alone is significantly lower and not higher than approximately 0.3 cases per 10,000 prescriptions [47,48]. Liver damage can be hepatocellular, cholestatic or mixed, with hepatocellular injury predominating among children [45] and mixed injury predominating among older patients [47,48]. This damage is generally mild, as it regresses completely as soon as the drug is discontinued in most cases. In a very low number of patients, however, significant functional and structural liver alterations may develop, leading to the need for hospitalization and transplant and an increased risk of death [49]. It has been reported that about 17% of all the DILI cases leading to hospitalization are associated with the prescription of AC [49]. Regarding the pathogenesis of hepatotoxicity, it is thought that, in most cases, it depends on genetic variations that lead to an immunological reaction (Table 2).

This conclusion is supported by evidence suggesting that the development of liver damage can be accompanied by other manifestations of immune-mediated injury, such as rash or eosinophilia [50]. Moreover, signs of hepatotoxicity may recur in a short time after re-exposure to the drug. Finally, most patients carry specific HLA alleles. Genetic studies have shown that AC hepatotoxicity is associated with many loci of the major histocompatibility complex, with the strongest effect being observed for DRB1*15:01-DQB1*06:02 [51]. An independent HLA Class I association has also been made with HLA-A*02:01 and with HLA-B*15-18. Subjects with these genetic variants were found to have an approximately three times higher likelihood of developing hepatoxicity when treated with AC than patients without treatment [52,53]. Practically, it is thought that the interaction of AC with these genetic variants leads to the formation of an immunogenic complex that is recognized by the immune system and evokes an immune reaction, thus causing hepatotoxicity.

However, some studies seem to indicate that AC-related DILI might depend on non-HLA variants, despite depending on alterations in immune system functions [52,53]. Associations with variants in two immune-related genes, namely the protein tyrosine phosphatase nonreceptor type 22 gene (*PTPN22*) and the endoplasmic reticulum aminopeptidase 2 (ERAP2), have been reported, despite having lower effect sizes than those seen with the HLA variants [51,51].

Despite the strong association between the presence of some genetic variants and the development of AC hepatotoxicity, genetic studies that aim to identify at-risk patients before the prescription of AC are not routinely performed. No suggestions regarding the implementation of population screening before AC use have ever been published. The risk of hepatotoxicity caused by AC is relatively low, and too many genetic variants are theoretically implicated in the determination of this adverse event. The implementation of screening in the general population seems too complicated and not cost effective. Despite this, some authors have prepared a polygenic score that includes all the five genetic variants previously reported and found to be able to identify subjects at risk of AG-related hepatotoxicity in the general population [52,53,54]. However, the authors themselves think that the use of this score is too complicated for current application and suggest that it is used only when acute severe liver disease of unknown origin is being assessed.

#### 2.2.2. Flucloxacillin

**Figure 2:**
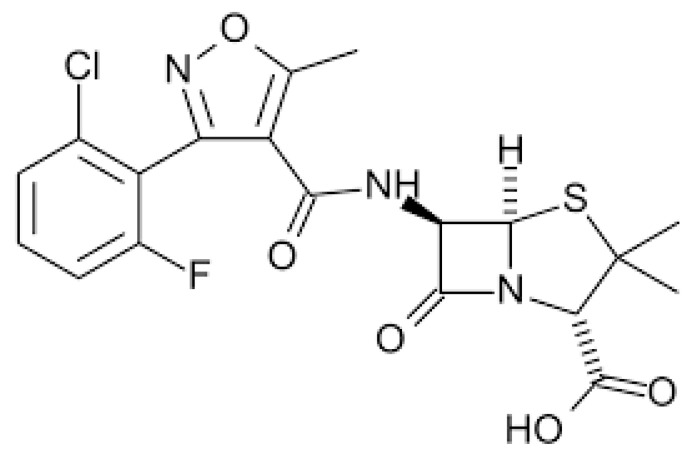
Chemical structure of flucloxacillin.

Flucloxacillin (FC) is an isoxazolyl penicillin, a group of antibiotics that includes flucloxacillin, oxacillin, cloxacillin, dicloxacillin and methicillin [55]. Figure 2 shows the chemical structure of FC.

All these drugs are beta-lactamase resistant and have been largely used to treat infections due to Gram-positive rods, mainly penicillin-resistant *Staphylococcus* spp. Presently, due to the emergence of methicillin-resistant *Staphylococcus* strains, the use of flucloxacillin and other isoxazolyl penicillins has been significantly reduced [56].

FC is generally safe and well tolerated, as adverse events following its administration are low in frequency and are mild and transient. However, the administration of FC can lead to the development of cholestatic hepatitis (Table 3), which occurs in 1–2 individuals per every 1,000 treated patients within 1 to 45 days of starting treatment [57]. This adverse event is relatively uncommon in children, as most cases have been diagnosed in patients older than 55 years [58]. In most cases, this liver disease tends to dissipate spontaneously in several months, even though the development of chronic vanishing bile duct syndrome is possible [59] and fatal cases have been described [60]. As for AC, immune pathogenesis is considered the basis for the development of liver disease caused by FC. It has been shown that more than 84% of patients with FC-associated hepatitis carry the HLA-B*57:01 allele, and that people with this genetic mutation have an 80 times greater likelihood of experiencing this adverse event [61,62,63]. Moreover, an association has also been seen with HLA-B*57:03 [62]. Interestingly, HLA-B*57 alleles have not been associated with hepatotoxicity induced by other isoxazolyl penicillins.

Despite the risk of patients receiving FC developing liver disease being well demonstrated, TDM is generally recommended only for the optimization of antibiotic exposure and the maximization of effectiveness, thereby potentially improving the disease outcome. In addition, the implementation of routine genetic testing before the initiation of therapy with this antibiotic is not routinely recommended. This complication is rare, and tests on the HLA-B*57:01 allele only offer a positive prediction in 0.12% of cases. It has been calculated that almost 14,000 patients would need to be screened to prevent a single case of severe liver disease [64]. However, it is suggested that liver function is carefully monitored during FC therapy, with the suspension of drug administration in the case of documented liver damage.

### 2.3. Antituberculous Drugs

Most patients, including children, suffering from tuberculosis are treated with a combination of multiple drugs; these are, most frequently, isoniazid (IS), rifampicin, pyrazinamide and ethambutol [65]. During treatment, generally between 6 weeks and 6 months after the start of drug administration [66], up to 20% of treated patients [67] develop signs of hepatotoxicity, with a lower frequency being observed among children [68,69]. Several factors, including age ≥60 years, female gender, a poor nutritional status and concomitant chronic hepatitis B infection, are associated with an increased risk of liver damage. However, genetic susceptibility seems to play a relevant role in this regard. In most cases, liver damage is limited to an asymptomatic, slight elevation in the concentration of serum transaminase in the liver that generally settles with the continued use of the drugs or disappears when the drugs are withdrawn. However, a subgroup accounting for approximately 1% of treated patients suffers from more severe drug-induced liver injury, with severe and prolonged transaminase elevation and relevant hepatocellular damage that can lead, in rare cases, to fulminant liver failure and death [70,71]. Although some cases of these adverse events have been ascribed to rifampicin [72] and pyrazinamide [73], IS is considered the most significant cause of liver damage in patients receiving antituberculous drugs [74]. Evidence suggesting that signs of hepatoxicity can be detected in patients of any age receiving isoniazid alone for prophylaxis strongly supports this conclusion [75].

#### Isoniazid

IS is metabolized in the liver by N-acetyltransferase 2 (NAT2) [76]. This enzyme assures the formation of acetyl isoniazid, which is in turn hydrolyzed to acetyl hydrazine and finally further acetylized to diacetyl hydrazine. IS and the first two metabolites are hepatotoxic, as they can lead to the formation of reactive oxygen species that cause cell necrosis and autoimmunity. Only the formation of diacetyl hydrazine assures liver integrity. Unfortunately, the hepatic NAT2 is polymorphic in humans, and the presence of mutations such as those detected in NAT2*7, NAT2*6 and NAT2*5 alleles can be associated with slow acetylation and the longer persistence of toxic metabolites [77]; this was evidenced in clinical studies enrolling patients with this metabolic condition and associated liver disease [78,79,80]. In a meta-analysis [81] of 24 studies involving a total of 1116 cases and 2655 controls, it was shown that the odds ratio (OR) of the *NAT2* slow acetylator genotype for liver damage was 3.18 (95% CI, 2.49–4.07); however, a difference according to ethnicity was observed. ORs of 3.32 [95% CI, 2.43–4.53), 2.96 (95% CI, 1.83–4.76), 6.64 (95% CI, 3.01–14.66) and 5.24 (95% CI, 2.18–12.60) were calculated for the slow acetylator genotype among East Asian, Indian, Middle Eastern and other ethnic populations, respectively. No association between liver damage and NAT2 mutations was evidenced in white people, but the low number of white patients enrolled in these studies may explain this finding. The increased risk of liver damage in patients carrying some *NAT2* mutations might suggest that the redosing of IS based on the genetic profiles of patients could maximize the efficacy of the treatment and minimize the risk of hepatotoxicity. The results of a study by Azuma et al. seem to confirm this supposition [82]. These authors reported that hepatotoxicity occurred in 78% of the slow acetylators receiving conventional treatment, while none of them experienced liver damage when the isoniazid dosage was halved.

Together with *NAT2* mutations, other genetic variants have been associated with IS hepatotoxicity. CYP2E1 is an enzyme that takes part in the metabolization of isoniazid as it oxidizes acetyl hydrazine to form N-hydroxy-acetyl hydrazine, which further dehydrates to yield acetyl diazine. From this, several toxic compounds develop [83]. Although with exceptions [84,85], studies have shown that subjects carrying the CYP2E1 c1/c1 genotype are 2.5 times more likely to develop hepatotoxicity when compared to those with other genotypes [86,87]. The risk of liver damage increased 7-fold when this genetic variant was associated with *NAT2* mutations, as the number of toxic metabolites was significantly increased [88]. An increased risk of liver damage was also reported in children with the CYP2E1*6 allele and *1A-*6-*1D haplotype [89].

The risk of liver damage also seems to be associated with *GSTM1* gene mutations [18]. This gene is included in a supergene family that encodes enzymes that play a significant role in the detoxification of several compounds via conjugation with glutathione, including drugs, environmental toxins and products of oxidative stress. The *GSTM1* null genotype is associated with an increased risk of liver damage in patients receiving IS. However, this risk seems limited to some specific ethnicities, as hepatotoxicity was reported in East Asian people but not in white and Indian populations [90].

Table 4 summarizes the genetic variants associated with IS-induced hepatotoxicity. Despite evidence suggesting that genetic variants, mainly *NAT2* mutations, can lead to an increased risk of IS toxicity and that genetic screening can prevent liver damage, no recommendations regarding systematic genetic screening have been made by health authorities.

In the USA, the Food and Drug Administration includes IS in the list of Pharmacogenetic Associations for which the Data Indicate a Potential Impact on Safety or Response [91]. However, although liver damage is included among the potential adverse events of the drug on its label, genetic mutations are not detailed and the need for pretreatment screening is not discussed. It is simply highlighted that the careful monitoring of liver function is required in patients receiving antituberculous drugs, including IS [92]. Once again, severe hepatotoxicity is rare, but more than one mutation is potentially associated with this condition; this extends the screening time and makes it more complicated and expensive. Universal pretreatment screening is also presently unthinkable in this case.

### 2.4. Sulfonamides

Sulfonamides were the first synthetic antimicrobial drugs introduced in clinical practice [92]. They were originally active against several Gram-positive and Gram-negative bacteria, and were largely used against a large number of bacterial diseases with satisfactory results. They are bacteriostatic agents that act by competitively inhibiting folic acid synthesis, which prevents the growth and proliferation of bacteria [93]. With the availability of antibiotics that are bactericidal, more effective, better tolerated and safe, the prescription of sulfonamides progressively declined. Most of them are now rarely used in clinical practice. The only exception is sulfamethoxazole (SMX), which, in combination with trimethoprim (TMP-SMX), is indicated and largely prescribed for prophylaxis and the treatment of several bacterial infections, including traveler’s diarrhea, urinary tract infections, and shigellosis; it is also included by the World Health Organization (WHO) in the list of essential medicines [94].

#### Sulfamethoxazole

Like all other sulfonamides, SMX use is associated with the development of allergic and hypersensitivity reactions in the general population, which globally occur in 3–8% of cases [95]. Immediate IgE-mediated reactions are generally mild or moderate and manifest as eosinophilia or exanthema. However, hypersensitivity reactions can be very severe, such as in the case of Stevens–Johnson syndrome or toxic epidermal necrolysis [95]. Several factors are associated with an increased risk of hypersensitivity reactions to SMX, including HIV positivity, long-term drug use and genetics [96]. It is thought that the most severe cases derive from SMX metabolites in patients carrying specific genetic mutations. SMX is metabolized by both *NAT1* and *NAT2* genes, and mutations in the *NAT2* gene can lead to a slow acetylator genotype status conditioning a more relevant formation of toxic metabolites such as hydroxylamine or nitroso compounds. Subjects with *NAT2* gene mutations receiving SMX were found to be more at risk of developing a hypersensitivity reaction than patients not receiving this treatment [97]. Hydroxylamine and nitroso compounds interact with tissue proteins to form haptenic structures that trigger hypersensitivity reactions. Moreover, SMX metabolites can activate T cells through the major histocompatibility complex, producing cytotoxic T lymphocytes that cause cell death and tissue damage. Finally, SMX itself can directly stimulate the immune system by activating T cell receptors via the major histocompatibility complex [98]. However, regardless of the mechanism implicated in hypersensitivity reactions, these are more common in subjects carrying particular HLA gene variations; among these, reactions involving the HLA-A29, HLA-B12, HLA-DR7, HLA-B44 and HLA A*11:01 gene variations are the most common [99]. Some authors have suggested that genetic screening should be implemented before the administration of SMX, but no official recommendations in this regard have ever been made [100]. It is only recommended that particular attention be paid to patients with previous hypersensitivity to sulfonamides. In this case, antibiotic replacement is the best option. Sulfonamides should only be administered when there is no other acceptable and effective treatment available [101].

## 3. Antibiotics for Which the Role of Genetics in Conditioning the Development of Adverse Events Is not Definitively Demonstrated

### 3.1. Linezolid

**Figure 3:**
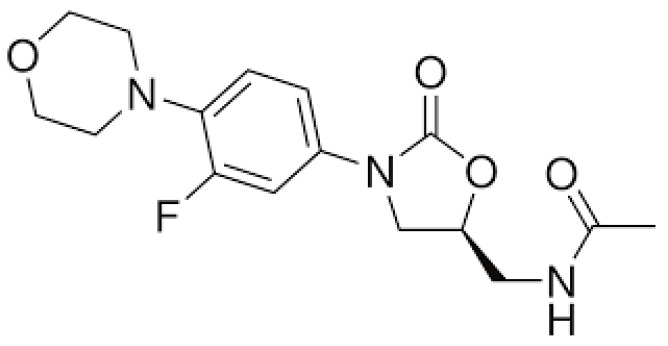
Chemical structure of linezolid.

Linezolid (LZ) is the first oxazolidinone antibiotic to be produced; this is a group of drugs recently developed to overcome some of the clinical problems strictly related to the emergence of bacterial resistance to antibiotics. Figure 3 shows the chemical structure of LZ.

LZ is effective against several Gram-positive drugs, including methicillin-resistant *Staphylococcus aureus*, and is indicated for the treatment of severe, life-threating conditions caused by these pathogens [102]. Moreover, as it is effective against multidrug-resistant or extensively-drug resistant *Mycobacterium tuberculosis*, LZ is included in the drug regimens used to treat patients infected with these resistant *M. tuberculosis* strains [103]. LZ exerts its antimicrobial activity via inhibition of bacterial protein synthesis in a way similar to that previously described for aminoglycosides. It binds to a site on the 23S ribosomal RNA of the 50S ribosomal subunit, thus preventing the formation of a functional 70S that is essential for the bacterial translation process [104]. Unfortunately, the long-term use of LZ is accompanied by the frequent development of several severe adverse events, including hyperlactatemia, lactic and metabolic acidosis, myelosuppression with thrombocytopenia and anemia, gastrointestinal disturbances and optic or peripheral neuropathy [105]. It is thought that, in most cases, these severe clinical problems depend, as in the case of AG, on the similarities between human and bacterial ribosomes. The inhibitory action exerted on protein synthesis and on bacterial ribosomes is extended to human ribosomes. On the other hand, in vitro studies have clearly shown that exposure to LZ significantly reduces the mitochondrial protein synthesis of mammalian cells [106,107]. Mitochondrial mutations may result in a predisposition to the development of LZ-related adverse events. Studies have shown that increased mitotoxicity and clinical symptoms can be found in patients harboring mtDNA haplogroup U, mutations in 12S rRNA or polymorphisms in the 16S rRNA sequence [108]. Moreover, polymorphisms in the *ABCB1* or *CYP3A* genes have been associated with the significant modification of LZ clearance, which may play a role in conditioning the efficacy and tolerability of the drug [109]. However, no definitive conclusions in this regard have been drawn and no health authorities have so far made recommendations related to the genetic control of patients receiving this drug [110]. 

### 3.2. Fluoroquinolones

Fluoroquinolones (FQs) are a group of antibiotics with a broad spectrum of activity and excellent pharmacokinetics [111]. This explains why they were, initially, frequently used. In recent years, however, the prescription of FQs has been significantly reduced due to evidence suggesting that they could cause rare but very serious, disabling and potentially irreversible adverse events involving the musculoskeletal, nervous and psychiatric systems of the body. This led health authorities to restrict the use of these drugs only to very severe infections, unless other antibacterial medicines commonly recommended for these conditions could not be used [112,113]. Levofloxacin, ciprofloxacin, moxifloxacin, ofloxacin, gemifloxacin and delafloxacin are the FQs most frequently prescribed to treat severe and complicated urinary tract infections, intraabdominal infections, skin and soft tissue infections, community-acquired and nosocomial pneumonia and bone and joint infections [114]. Additionally, FQs such as moxifloxacin, gatifloxacin and levofloxacin are seeing increased off-label usage in the treatment of drug-resistant tuberculosis or cases of intolerance to other antituberculosis drugs [115]. The routine use of systemic FQs should be avoided in children due to the already reported potential risk of musculoskeletal toxicity. However, their off-label use, especially in children with cystic fibrosis or tuberculosis due to resistant bacteria, is relatively common [111].

It has been suggested that, at least in part, FQ-related adverse events are related to genetic variants that condition significant variations in drug disposition. FQs are substrates of the multiple ATP-binding cassette (ABC) superfamily of active transporters, which play a critical role in conditioning the passage of these drugs into tissues and across the blood–brain barrier [116,117]. In a case report, it was evidenced that a patient who had developed generalized seizures after treatment with levofloxacin carried polymorphisms of the efflux transporter genes *ABCB1* and *ABCG2*, which code for P-glycoprotein and breast cancer resistance protein (BCRP), respectively. This could have conditioned the reduced activity of both these proteins and the increased passage of levofloxacin across the blood–brain barrier, causing seizures [118]. Polymorphisms of the *ABCB1* gene and of the UDP glucuronosyltransferase family 1 member A1 (*UGT1A1*) gene were found in a small group of patients experiencing the reduced absorption of moxifloxacin, further supporting the hypothesis that genetics may condition the disposition of FQs and the development of unexpected adverse events [119]. This suspicion is further confirmed by the result of a study involving a second group of patients receiving moxifloxacin [120]. In this case, it was found that a significant increase in drug blood levels could be found in subjects carrying the -1187G>A variant in the solute carrier organic anion transporter family member 1B1 gene (*SLCO1B1*). This suggests that increased blood concentrations of the antibiotic could explain the prolongation of the QT interval and the other cardiac arrhythmias frequently reported among FQ-related adverse events. Although interesting, these findings do not definitively demonstrate a clear relationship between specific genetic variations and the development of some FQ-related adverse events. Further studies are needed to definitively clarify these problems.

### 3.3. Macrolides

**Figure 4:**
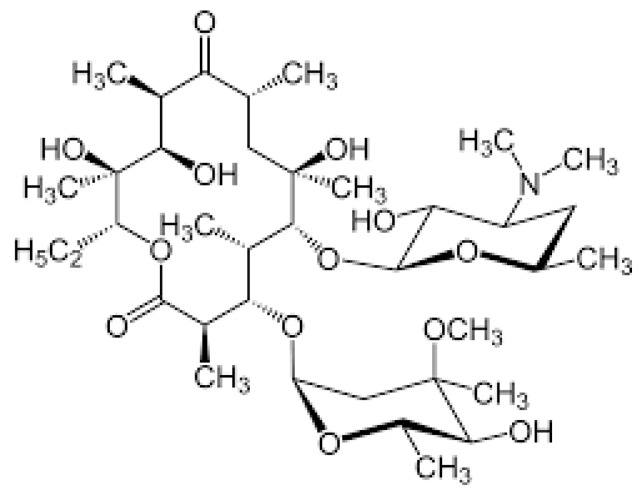
Chemical structure of macrolides.

Macrolides (MCs) are a group of antibiotics characterized by a large lactone ring, which can vary from 12 to 16 atoms, with one or more sugar chains attached (Figure 4).

The most used MCs are erythromycin (ER), clarithromycin (CL) and azithromycin (AZ). ER and CL are 14-membered macrolides, whereas AZ is a 16-membered drug [121]. Like several other antibiotics, MCs act via inhibition of bacterial protein synthesis. They interfere with ribosomal activity, binding to the bacterial 50S ribosomal subunit and preventing the translation of mRNA. Bacterial protein synthesis is consequently inhibited [122]. MCs are largely effective against several bacteria pathogens, including *Streptococcus pyogenes*, *Streptococcus pneumoniae*, *Hemophilus influenzae*, *Bordetella pertussis* and atypical bacteria. For this, they are considered an optimal solution for the treatment of respiratory infections and skin and soft tissue infections, particularly when penicillin cannot be used. Moreover, due to their activity against Helicobacter pylori, MCs have been introduced into a combination therapy for infections caused by this pathogen. Unfortunately, in recent years, a significant number of previously highly sensitive bacteria, especially *S. pyogenes* and *S. pneumoniae*, have developed resistance to MCs, and experts have recommended that these agents are prescribed only when the sensitivity of the infecting pathogen has not been previously established or is strongly suspected [123]. However, MCs have continued to be used in the treatment of atypical bacterial infection and Helicobacter pylori infections [124]. Generally, MCs are safe and well tolerated, although some relevant adverse events occasionally occur. The prolongation of the QT and QTc interval in the cardiac cycle, potentially favoring the development of cardiac arrhythmias like torsades de pointes, ventricular tachycardia, and ventricular fibrillation, is the most common adverse event reported [125].

Several reports seem to suggest that genetic variations in MC-metabolizing enzymes and transporters can significantly modify the disposition of MCs [126]; this is evidenced by some of the examples reported below. However, none of these studies indicate whether these genetic variants lead to an increase in the incidence of adverse events.

ER and CL are metabolized by cytochrome P450 3A4 (CYP3A4) and are transported by MRP2, which is encoded by the *ABCC2* gene. Studies have shown that variations in CYP3A4 may affect the metabolism of ER. This is suggested by the evidence suggesting that people of Asian descent exhibit less CYP3A4 activity than white people, and that these people have a 65% higher bioavailability of ER at the same dose [127]. Polymorphisms of the *ABCC2* gene can lead to a reduced MRP2 function that in turn causes an increased permanence of the drug in the hepatocytes [128]. Moreover, as ER is also transported by OATP1B1 encoded by the *SLOC1B1* gene, polymorphisms of this gene can modify ER transport. In vitro studies have confirmed this supposition, with the transport of the drug being significantly reduced by cells with the OAT1B1*5 variant compared to those without. Moreover, in animas patients with the OAT1B1*5 variant, the metabolism of ER was significantly impaired [129].

AZ is transported by P-glycoprotein and the *MRP2* gene. Polymorphisms of *ABCB1* can influence the concentration of AZ in the blood, with values that are higher in subjects carrying the 2677TT/3435 TT genotype than in those with the 2677GG/3435CC genotype [8,130].

## 4. Conclusions

The genetic screening of patients with specific genetic polymorphisms related to drug toxicity, as in the case of allopurinol, carbamazepine and abacavir, has been found to be extremely effective in minimizing the incidence of drug-related adverse events in at-risk subjects [18]. However, regarding antibiotics, defined associations between genetic variations and specific adverse events are very few; to date, none have led to the implementation of a systematic process for the screening of patients that must be treated with a given drug in order to select those at risk of specific adverse events. On the other hand, in most cases, more than one variation may be implicated in the development of an adverse event, and this could be considered a limitation in the planning of a systematic screening process. Moreover, at present, the methods used to establish whether a patient carries a “dangerous” genetic mutation require too much time, and waiting for the results of the test can be deleterious for those patients urgently needing therapy. In addition, it is not clear which molecules manage antibiotic-related adverse events on the basis of the pharmacogenomic results.

Both pharmacogenomics and TDM offer potential benefits regarding the optimization of antibiotic therapy, but they also have limitations and challenges that need to be addressed if they are to be effectively implemented in clinical practice (Table 5). Integrating these approaches into routine patient care requires a careful consideration of their clinical utility, cost effectiveness, and ethical implications, along with continued research to enhance their evidence base and expand their applicability to a wider range of antibiotics and patient populations.

Further studies are needed to definitively confirm which genetic variations are responsible for an increased risk of a well-defined adverse event. Moreover, when reliable information is collected, it is essential that specific point-of-care genetic testing is made available. Only in this way will the universal screening selection of patients at risk of severe adverse events following the administration of particular antibiotics be possible. 

## Figures and Tables

**Table 1:** Mutations in the mitochondrial gene *RNR1* (MT-RNR1) associated with aminoglycoside (AG)-related deafness.

Mutation	Prevalence in Patients with AG-Related Deafness
m1555A>G	5–33%
1095 T>C	<5%
1494 C>T	<5%

**Table 2:** Genetic variations associated with idiosyncratic drug-induced liver injury (DILI) due to amoxicillin–clavulanic acid (AC).

Variation	Increase in DILI Risk
DRB1*15:01-DQB1*06:02	x3
HLA-A*02:01	x3
HLA-B*15-18	x3
*PTPN22* gene	x2
*ERAP2* gene	x2

**Table 3:** Genetic variations associated with cholestatic hepatitis due to flucloxacillin (FC).

Variation	Increase in Risk of Cholestatic Hepatitis
HLA-B*57:01	X80
HLA-B*57:03	x37

**Table 4:** Genetic variations associated with isoniazid (IS)-induced hepatotoxicity.

Variation	Increase in Risk of Hepatotoxicity
NAT2*7, NAT2*6 andNAT2*5	x3–7
CYP2E1 c1/c1 genotype	x2.5
CYP2E1*6 allele	x2
CYP2E1*1A-*6-*1D haplotype	x2
*GSTM1* null genotype	x2

**Table 5:** Pros and cons of pharmacogenomics and therapeutic drug monitoring for optimizing antibiotic therapy.

Test	Pros	Cons
Pharmacogenomics	**Personalized Medicine:** Pharmacogenomics allows for the customization of antibiotic therapy based on an individual’s genetic makeup. This can lead to more effective and safer treatment by targeting the specific genetic factors affecting drug metabolism and response. **Reduced Adverse Effects:** By identifying genetic variations that affect drug metabolism and response, pharmacogenomics can help prevent adverse drug reactions and toxicity, leading to safer antibiotic use. **Optimized Drug Selection:** Pharmacogenomic testing can guide clinicians in selecting the most appropriate antibiotic for a particular patient, based on their genetic profile. This can enhance treatment efficacy and reduce the risk of treatment failure. **Improved Antibiotic Stewardship:** By tailoring antibiotic therapy to individual patients, pharmacogenomics can contribute to antibiotic stewardship efforts by minimizing the unnecessary use of broad-spectrum antibiotics and reducing the risk of antibiotic resistance.	**Cost:** Pharmacogenomic testing can be expensive, which may limit its widespread adoption, especially in resource-constrained healthcare settings. **Complexity:** Interpreting pharmacogenomic test results and integrating them into clinical decision making can be complex and time consuming for healthcare providers. **Limited Evidence:** While pharmacogenomics holds promise for optimizing antibiotic therapy, the evidence supporting its clinical utility in this context is still emerging, and more research is needed to fully understand its impact on patient outcomes. **Ethical and Privacy Concerns:** Pharmacogenomic testing raises ethical and privacy concerns related to the storage and use of genetic information, as well as potential implications for insurance coverage and employment discrimination.
Therapeutic drug monitoring	**Individualized Dosage Adjustment:** TDM allows for the monitoring of antibiotic concentrations in the blood, enabling clinicians to adjust dosage regimens to achieve optimal therapeutic levels for individual patients. **Maximized Efficacy:** By ensuring that antibiotic concentrations remain within the therapeutic range, TDM can maximize treatment efficacy and reduce the risk of treatment failure and the development of antibiotic resistance. **Prevention of Toxicity:** TDM helps prevent antibiotic-related toxicity by monitoring drug levels and minimizing the risk of supra-therapeutic concentrations that can lead to adverse effects. **Real-Time Feedback:** TDM provides clinicians with real-time feedback on drug levels, allowing for timely adjustments to dosage regimens and enhancing patient safety.	**Resource Intensive:** TDM requires specialized equipment and expertise for sample collection, analysis and interpretation, which can be resource intensive and may not be readily available in all healthcare settings. **Limited Availability of Assays:** Not all antibiotics have commercially available assays for TDM, which limits its applicability to a subset of antibiotics and may restrict its utility in clinical practice. **Timing Issues:** TDM may not always provide timely feedback for adjusting antibiotic therapy, especially in acute care settings where rapid decision making is crucial. **Interpatient Variability:** Interpatient variability in drug metabolism and response can complicate the interpretation of TDM results and may necessitate individualized dosing strategies based on factors other than drug levels alone.

## Data Availability

Not applicable.
